# Intestinal Obstruction from Impaction of a Gall-Stone in the Ileum

**Published:** 1887-08

**Authors:** Bryan Holme Allen


### CASE OF INTESTINAL OBSTRUCTION FROM IMPACTION OF A GALL-STONE IN THE ILEUM.

I WAS called to Mr. C. E., aged fifty-seven, on Sept. 20, 1886, on account of constant sickness followed by faintness. On inquiry I obtained the following history. He had enjoyed fairly good health, but had had an attack of typhoid fever twenty-eight years ago. He was always disposed to be constipated, but by the free use of aperient medicine had kept his bowels acting. He had been a somewhat free liver, and for the last few years had suffered from business anxieties. On July 14th of this year (1886) he was seized with severe vomitıng and acute pain in the epigastrium and right hypochondrium, followed by jaundice; the pain was intense for three or four days; the urine high colored. I could obtain no definite information about the motions ; he was supposed to have passed a gall-stone, though nothing was ever seen in the motions. In a month's time he returned to business, feeling fairly well, but not losing the sense of uneasiness in the right hypochondrium. His bowels were more obstinate than ever, acting for the last time on Sept. 13th. On the same day he was seized with frequent vomiting of green bilious matter. On the 17th he consulted a physician in London, who carefully examined him, and finding nothing in the abdomen, attributed the vomiting to gastric catarrh, and advised him to come to Hastings, which he did on the 20th. On that day I found him complaining of great faintness, and of some pain in the stomach, mostly at the epigastrium, but to a less degree in the region of the cæcum and descending colon, with moderate tenderness in these latter regions. He was vomiting frequently, the vomited matter being very green, of an acid, disagreeable, but not at all feculent smell. His bowels had not acted for a week, nor had he passed flatus per anum. No tumor or special resistance could be anywhere felt in the abdomen. There was no abdominal distension, and no visible movement of the coils of intestine. The pulse was large, soft and quick. He was sent to bed, moderate doses of opium administered, and an injection ordered, which failed of effect, probably from imperfect administration. On Sept. 21st, 22nd and 23d he had an injection each day of three or four pints of soap-and-water, which on each occasion brought away some green, offensive, solid, but not hard, lumps, after which the abdominal tenderness disappeared and the pain and sickness greately diminished, the character of the vomited matter remaining unchanged. He was taking ten minims each of the solution of morphia and tincture of belladonna every four hours, or oftener if required. His diet consisted of half a pint of milk and a little beef-tea in twenty-four hours; a beef-tea enema was given every four hours. On the 24th he was ordered a five-grain cathartic pill (U.S. Pharm.) at bed-time, followed in the morning by an injection through a long flexible tube. On the 25th two pills of the same were given, also followed by an injection, which brought away motions of the same character as before. The vomiting varied from once to three times in the twenty-four hours, but was becoming more distinctly unpleasant. Very little pain; no tenderness or distension; no wind passed per anum. On the 26th and 27th the injections were repeated, but no action followed, and on the evening of the latter day the vomiting was distinctly feculent. There was no distension of the abdomen. On the 28th Dr. Arthur Gamgee saw him with me, and suggested the employment of electricity, Chloroform was given, and a current from a large primary coil was used, being passed from one side of the abdomen to the other, and afterwards through the rectum, but although violent action of the abdominal muscles was produced, no other effect resulted. Nothing definite could be felt in the abdomen under chloroform, but Dr. Gamgee thought that he felt greater resistance in the region of the ascending colon.

The intestinal obstruction had now been complete for sixteen days. Some fæcal matter had been brought away by the injections, but this evidently came from below the obstruction. No flatus had been passed since September 13th, when the symptoms of obstruction set in. After careful consideration of the symptoms I now came to the conclusion that the case was one of impacted gall-stone in the ileum, and that there was little or no hope of relief except by operation. I was led to this diagnosis by the following points. The invasion was sufficiently sudden to bring it within the class of acute obstructions, and there was a total absence of distension of the abdomen. I therefore excluded all diseases of the great intestine, including volvulus. At the same time, the onset was not sufficiently sudden for an internal strangulation or twist of the small intestine. Moreover, there was a total absence of collapse, the vomiting had never been severe, it was not stercoraceous until the fourteenth day, and the pain was far from acute. The absence of blood or mucus in the evacuations brought away by the enemata, the completeness of the obstruction, and the fact that no tumor could be felt, even under chloroform, seem to negative the idea of acute intussusception. It was quite certain that there was no peritonitis, either chronic or acute. All these conditions being excluded, obstruction from a foreign body impacted in the small intestine, either at its narrowest part near the lower end of the ileum, or in some part narrowed by disease, seemed the only probable diagnosis. That the foreign body was a gall-stone I concluded from the history of hepatic colic ten weeks before, accompanied by little, if any, jaundice, and followed by uneasiness in the region below the liver, continuing up to the time of the attack.

On Sept. 29, at 1 P. M., Mr. Marcus Beck saw the case with me, and, agreeing in the diagnosis, proceeded to open the abdomen, assisted by Dr. Gamgee, Mr. Brodribb and myself. Mr. Beck has furnished me with the following account of the operation. The patient, having been placed on a table, was covered with a mackintosh sheet having an oval opening in the middle (an ovariotomy apron). The skin was then carefully cleaned with a 1 in 20 solution of carbolic acid. Ether having been administered a spray of carbolic acid was turned on. An incision was then made (about three inches in length) in the middle line, the middle of the wound being midway between the umbilicus and the pubes. The abdomen being opened, a coil of distended small intestine showed in the wound. The hand was passed at once into the right iliac fossa, with the intention of feeling for the cæcum, in order to ascertain whether it was distended or empty. Before reaching the cæcum, but close to it, a hard mass was felt in the coil of the small intestine. This was at once drawn up into the wound and out of the abdomen. It was then seen that the gut was completely plugged by a barrel-shaped body of great density. The gut was apparently healtly; its surface was shiny and perfectly free from any sign of inflammation. An attempt was made to push the foreign body forwards, the direction in which it should go being easily recognized by the distended condition of the intestine above it, while below the gut was empty; it would not move with any amount of force which it seemed justifiable to use. It was determed, therefore, to cut it out by a longitudinal incision in the intestine. The opening in the abdomen was carefully plugged with sponges which had been rung out of a warm solution of carbolic acid (1 in 40), and the coil of intestine was laid upon a flat sponge; a small hole was then made in the mesentery close to the gut, immediately above the foreign body; through this a piece of india rubber drainage-tube was passed and drawn sufficiently tight to occlude the gut, and clamped with a pair of forci-pressure forceps. In this way the escape of fæces from above was effectually prevented. A longitudinal incision was then made at the side opposite the attachment of the mesentery. It was about one inch and three-quarters in length, and was of sufficient size to allow of the escape of the foreign body without bruising the tissues of the gut. A small quantity of very dark fæcal matter escaped, which was immediately received on a sponge. The mucous membrane was intensely injected and terribly swollen. But little blood flowed from the incision. The gut having been cleaned, the edges of the wound were brought together by interrupted Lembert's sutures. Fine common sewing needles were used (No. 7), threaded with fine carbolized silk. The needle was inserted about a quarter of an inch from the edge of the incision, penetrating the serous and muscular coats only, and brought out about one-sixth of an inch from the edge; then inserted on the opposite side about one-sixth of an inch from the edge, and brought out a quarter of an inch. About a dozen stitches were required and were inserted. When all the necessary sutures were inserted they were tied. The closure seemed perfect, the mucous coat being turned in and the serous from each side brought well in contact. The piece of india rubber tube was now removed; it had made rather a deep impression in the gut, showing the swollen condition of the bowel. The impression began to disappear immediately, the circulation being rapidly restored in the pale part. The incision was then minutely examined to see if anything leaked from it, and nothing was detected. The coil of intestine was then carefully cleaned, the sponges plugging the abdominal wound removed, and the gut replaced in the abdomen. During the whole operation no blood or other foreign matter had escaped into the peritoneal cavity. It was not necessary, therefore, to clean out the peritoneum by sponging or washing. The external wound was then closed in the usual way. The anæsthetic was admirably administered by Mr. Brodribb, so that at no time was there the slightest trouble from any movement on the part of the patient. There was but little shock. The wound was dressed with sal alembroth gauze, covered by a layer of sal alembroth wool. The foreign body was found to be a large gall-stone. It was somewhat the shape of a conical bullet, the advancing end being slightly conical, the opposite end flat. It was marked at its base by a smooth, slightly concave facet, evidently formed by contact with another stone in the gall bladder. The rest of the stone was rough on the surface, and glistening crystals of cholesterine could be seen upon it. This roughness must have considerably impeded its passage down the intestine. It measured 3⅞ in. in circumference, 1¾ in. in length. It weighed almost exactly an ounce. The patient rallied well from the operation. A dose of morphia was administered hypodermically, and at 9 P. M. the pulse was 100, and of good force; there was no pain or sickness, and the patient seemed to be doing well, but on the following morning the pulse had risen to 120, the countenance was anxious, there was slight distension and some tenderness of the abdomen, but no sickness. He died at 3 P. M., exactly twenty-five hours after the operation. No post-mortem examination could be obtained.

In the treatment of this case I was led to go somewhat beyond the orthodox way of feeding in intestinal obstruction by the willingness of the patient to take food, by the fact of its being retained several hours, and from its not, as far as I could see, in the smallest degree promoting vomiting, and perhaps also from having less faith in the value of nutritive enemata than some have. The medical treatment consisted of opium and belladonna—the doses, in consequence of the moderate amount of pain, being comparatively small—enemata, and, when they no longer acted, aperients. I was led to try aperients from the recollection of a case I had some years ago, when the combined use of aperients and enemata resulted in the passing, after ten days' absolute constipation, of a stone so large that its passage through the anus caused great suffering.

The following points seem worthy of further consideration in this case: 1. What was the cause of death? Unfortunately this question must remain doubtful. He did not die from shock, for he rallied well from the operation. Every precaution was taken to ensure cleanliness, no carbolic lotion except that upon the hands entered the abdominal cavity, the stitches seemed efficiently to close the gut, well carbolized silk was used, and yet from the period of death it seemed most probable that peritonitis was the cause. The gut, though not actually inflamed at the time of operation, was much congested and swollen. Most probably inflammation developed in the damaged gut after the operation, and in the weak state of the patient proved rapidly fatal. 2. When was the stone impacted? This could not be accurately ascertained. From the size of the gut and the situation in the abdomen it was probably in the lower part of the ileum, not far from its end. That it was the ileum is certain from the absence of the valvulæ conniventes in the part opened. 3. Was the operation necessary, or would it have been safer, having made the diagnosis, to trust to nature? This question is extremely difficult to answer. I find in the Index Catalogue of the library of the Surgeon-general, U. S. A., references to thirty-nine cases of impacted gallstone. Of these I have been able, with the limited time at my command, to refer to only fourteen. Among them, I find, is a stone measuring 3½ in. passed safely after twelve days' obstruction. In another case there was a stone of a little over three inches, but there is no evidence that it passed down the small intestine; it probably ulcerated from the gall-bladder into the colon. Fagge also mentions one measuring 1 1-10 in. in diameter, which passed after seven days' obstruction. On the other hand, I found seven stones, proving fatal, which measured less than that from my patient; the smallest had a transverse circumference of only 2¼ in., and yet it blocked the lower end of the jejunum, and caused death after fæcal vomiting. It seems, therefore, that although it is possible for a patient to pass stones of this size through the ileo-colic valve, very few succeed in doing so, and probably the percentage of recovery would be greater if operation were resorted to in every case as soon as the diagnosis is made. Unfortunately, however, the diagnosis is very rarely made at all. In almost all the cases I have met with, their nature was only cleared up at the post-mortem examination. I can find no case but this in which the stone has been removed by operation. The disease is far more common in women than in men. In the fourteen cases I have been able to refer to only two were men.—*Bryan Holme Allen, M. D., in the Lancet.*

